# Bringing the Laboratory Home: PANDABox Telehealth-Based Assessment of Neurodevelopmental Risk in Children

**DOI:** 10.3389/fpsyg.2020.01634

**Published:** 2020-07-28

**Authors:** Bridgette L. Kelleher, Taylor Halligan, Nicole Witthuhn, Wei Siong Neo, Lisa Hamrick, Leonard Abbeduto

**Affiliations:** ^1^Department of Psychological Sciences, Purdue University, West Lafayette, IN, United States; ^2^Department of Psychiatry and Behavioral Sciences, MIND Institute, University of California, Davis, Davis, CA, United States

**Keywords:** telehealth, remote assessment, down syndrome, neurogenetic syndromes, heart rate, autism

## Abstract

**Background:**

Advances in clinical trials have revealed a pressing need for outcome measures appropriate for children with neurogenetic syndromes (NGS). However, the field lacks a standardized, flexible protocol for collecting laboratory-grade experimental data remotely. To address this challenge, we developed PANDABox (Parent-Administered Neurodevelopmental Assessment), a caregiver-facilitated, remotely administered assessment protocol for collecting integrated and high quality clinical, behavioral, and spectral data relevant to a wide array of research questions. Here, we describe PANDABox development and report preliminary data regarding: (1) logistics and cost, (2) caregiver fidelity and satisfaction, and (3) data quality.

**Methods:**

We administered PANDABox to a cohort of 16 geographically diverse caregivers and their infants with Down syndrome. Tasks assessed attention, language, motor, and atypical behaviors. Behavioral and physiological data were synchronized and coded offline by trained research assistants.

**Results:**

PANDABox required low resources to administer and was well received by families, with high caregiver fidelity (94%) and infant engagement (91%), as well as high caregiver-reported satisfaction (97% positive). Missing data rates were low for video frames (3%) and vocalization recordings (6%) but were higher for heart rate (25% fully missing and 13% partially missing) and discrete behavioral presses (8% technical issues and 19% not enough codable behavior), reflecting the increased technical demands for these activities.

**Conclusion:**

With further development, low-cost laboratory-grade research protocols may be remotely administered by caregivers in the family home, opening a new frontier for cost-efficient, scalable assessment studies for children with NGS other neurodevelopmental disorders.

## Introduction

Accurately assessing developmental skills in children with NGS has become a pressing need given recent advances in clinical trials and the lack of appropriate outcome measures suitable for children with IDD (Budimirovic et al., 2017; Grieco et al., 2019). For example, low-quality outcome measures have been blamed, in part, for the recent failure of drug trials in fragile X syndrome (Erickson et al., 2017), and federal agencies have released specific funding announcements focused on outcome measure development for these populations (e.g., NIH PAR 18-039; “Outcome Measures for Use in Treatment Trials of Individuals with IDD”). Although a number of novel measures are being developed and modified to address these needs, many of the most promising assessment tools – including clinical and neuroimaging approaches – require in-person administration by a trained examiner. Regular in-person assessments are necessary to assess patient safety during trials. However, supplementing these visits with remotely administered outcome assessments may reduce the number of clinic visits required for trials, reducing both cost and participation burden for patients. These alternate assessment approaches may particularly benefit patients with low-incidence NGS who are often widely geographically distributed and do not live near trial sites.

To address these challenges, a number of groups have begun developing portable or telehealth-based outcome measures that are suitable for children with IDD, such as novel behavioral language tasks that can be administered via telehealth (Nelson et al., 2018). However, to date, most of these efforts have relied on *clinical* (e.g., parent interviews) and *behavioral* (e.g., video observations) methods that are feasible to implement using commercially available platforms. Telehealth has been less frequently used to collect data using what we describe as *spectral* methods–traditionally laboratory-based assessment techniques that capture behaviorally anchored, high-density spatial or temporal characteristics of participant responses not detectable through observation alone. Spectral methods such as eye tracking, facial coding procedures, and biosensor assays commonly hailed as the most objective and sensitive metrics for monitoring acute changes over the course of clinical trials (Berry-Kravis et al., 2013; Jeste et al., 2015). These methods are particularly well suited to IDD populations because assessments can be designed to require minimal-to-no voluntary responses from participants and do not rely on standardized scores that often pose challenges for children with severe developmental delays. However, standardized protocols for collecting spectral data via telehealth are lacking, and commercial-grade biosensors have performed poorly in recent largescale outcome measures studies (Ness et al., 2019). The present study proposes a feasible and low-cost solution to this challenge – PANDABox – a caregiver-facilitated, remotely administered assessment protocol for collecting integrated, laboratory-grade outcome measures data in NGS and other clinical populations.

### The Promise of Telehealth-Based Outcome Measures

In the broader field of neurodevelopmental disorders, telehealth-based clinical research has rapidly increased over the past decade, with applications ranging from parent-facilitated interventions (Vismara et al., 2013; Nelson et al., 2018) to autism screening (Parmanto et al., 2013; Talbott et al., 2019) to improving clinical access for rural populations (Lindgren et al., 2016). This surge parallels broader movements toward big data, wearable devices, and home-based healthcare (Piwek et al., 2016; Raugh et al., 2019). For example, wearable devices have been developed to automatically assay a range of variables including sleep quality, language production, physiological arousal, and eye movements (Cabibihan et al., 2017). Computational approaches are being optimized to process these rich, high-density signals in new ways, including by use of machine learning algorithms (Kosmicki et al., 2015; Hazlett et al., 2017) and citizen science or crowdsourced coding (Dickinson et al., 2010; Casler et al., 2013). These advances align with recent recognition of potential benefits of electronic patient reported outcomes (ePRO) in clinical trials (Byrom et al., 2018a) and emerging recommendations for best practice and use for biosensors and wearable devices (Byrom et al., 2018b).

Despite these advances, a notable gap remains: when implemented outside of controlled laboratory settings, biosensors and other spectral measures are rarely integrated with observed behavioral data in studies of neurodevelopmental disorders. Indeed, most clinical studies using commercial-grade wearable devices focus on broad patterns of daily activity, such as number of vocalizations (Reisinger et al., 2019) or patterns of heart rate (Ness et al., 2019). Other fields have linked these types of data to daily events or patient-reported experiences, such as through environmental momentary assessment (Raugh et al., 2019). However, even within these largely adult-focused studies, the specific antecedents of change are rarely objectively measured or standardized. This uncontrolled variability can interfere with interpretation of patient outcomes, as it is often unclear whether and how contextual factors influence behavior. In part, this limitation reflects that most commercially available biosensors do not produce the level of high-quality, temporally precise raw data necessary to conduct standard laboratory tasks in naturalistic settings. For example, heart rate decelerations during sustained attention may be a useful marker of cognitive engagement in high-risk infants (Tonnsen et al., 2018), however, quantifying these decelerations requires highly precise data that can be accurately aligned with the timing of visual stimuli. Other spectral outcomes such as event-related potentials require even greater temporal precision, as responses occur within milliseconds of stimulus onset. These constraints are typically addressed in controlled laboratory settings via highly trained staff, sophisticated equipment, and elegant data processing pipelines. However, this level of quality control and integration is often not feasible using commercially available telehealth platforms. As such, many promising paradigms for monitoring development and change in IDD populations remain restricted to laboratory or clinical settings.

### The Need for a New Paradigm

Developing a paradigm for collecting laboratory-grade, integrated spectral data remotely has potential to rapidly expand the quality and scope of outcome measures available for clinical trials. It is also possible that this new paradigm could improve trial validity. Indeed, clinic-based studies likely over represent families whose children lack behavioral or medical symptoms that would interfere with travel, such as aggression or inflexibility to changes in routine. Clinic-based studies also tend to favor families with resources needed for travel (funding, flexible careers, and childcare) and those who are comfortable interacting with providers in medical or academic settings. These biases particularly affect individuals from disadvantaged backgrounds, compromising generalizability and cultural validity of findings (Nielsen et al., 2017). Telehealth may improve representation of diverse patient populations by reducing geographic barriers and connecting patients to providers with expertise in aspects of diversity important to the patient (Ashburner et al., 2016). However, it is also possible that telehealth could, in many cases, introduce new constraints that compromise outcomes for underserved populations. For example, one study of rural families noted that participants expressed concerns about technical challenges (e.g., slow internet connection and lack of experience) and preferred that telehealth be used to supplement rather than replace face-to-face services (Ashburner et al., 2016). Thus, although telehealth has substantial promise in promoting representation in clinical research, it is important to consider multiple facets of access to ensure telehealth reduces–rather than amplifies–disparities.

In addition to improving trial validity, telehealth-based outcome measures may also more broadly benefit the power, rigor, and reproducibility of clinical science. The “replication crisis” heavily discussed in many fields commonly cites small, underpowered samples as a major source of error in clinical science (Open Science and Collaboration, 2015; Frank et al., 2017). Indeed, underpowered studies are likely to produce both imprecise and inflated estimates of effect sizes in neurodevelopmental research (Lombardo et al., 2019). However, for many clinical samples, publishing small samples is a critical step to larger, high-powered, and more resource-intensive studies. For rare NGS, small, geographically distributed samples are simply a reality. Telehealth has the potential to ease this challenge by providing opportunities to enhance the size (number of accessible participants) and density (more assessments per participant) of longitudinal surveillance, such as by supplementing clinic-based assessments (e.g., gold-standard diagnostic interviews and neuroimaging) with repeated remote assessments of related constructs. Telehealth may also conserve costs, facilitating larger sample sizes. For example, telehealth-based autism intervention has been estimated to reduce costs of clinic-based therapies by as much as 64% (Lindgren et al., 2016) and costs associated with other medical conditions in non-ASD populations by 17–75% (Dinesen et al., 2016). Thus, telehealth-based outcome measures may enhance rigor and reproducibility by facilitating wider-reaching, lower-cost outcome assessments.

### PANDABox as a Solution

There is a strong case for leveraging telehealth to monitor outcomes of children with NGS enrolled in clinical trials. However, to date, no standardized, open-science option is available to collect integrated, spectral data. To meet this need, we developed PANDABox, a telehealth-based, caregiver-facilitated, customizable protocol for monitoring early developmental features in NGS populations. In this paper, we describe the process of developing PANDABox using a sample protocol that integrates multiple levels of measurement–clinical, behavioral, and spectral–to assess early developmental features associated with atypical development in infants and toddlers. We then present pilot data from a small cohort of geographically diverse children with Down syndrome who completed PANDABox. We chose this population because Down syndrome is associated with atypical development across a variety of domains, and children with Down syndrome often exhibit elevated rates of ASD-associated behaviors that could be particularly salient targets for clinical trials (Warner et al., 2017; Moore et al., 2019). Similar to NGS, Down syndrome is also associated with a number of medical comorbidities (e.g., heart problems, low muscle tone, and strabismus) that we wanted to ensure PANDABox could accommodate. We specifically focused on three indicators of feasibility: (1) logistics and cost, (2) caregiver implementation fidelity and self-reported assessment experience and (3) child engagement and data quality. We conclude by discussing next steps for PANDABox, with attention toward current applications, scalability, and open science approaches. Collectively, this work sets the foundation for leveraging telehealth to bring the full laboratory home–improving the power, density, and representativeness of clinical research in ASD and other neurodevelopmental populations.

## Materials and Methods

PANDABox is a home-based laboratory assessment that is facilitated by a caregiver with live, remote support from a centralized support assessor. PANDABox is a modular battery, meaning tasks can be modified to suit a variety of research questions and participant samples. The task battery for the present study includes an initial set of activities that were selected based on the following logistical- and research-motivated criteria:

(1) Empirical relevance for atypical outcomes across a variety of developmental domains, including attention, language, motor, and atypical behaviors (e.g., “red flags” for ASD).
(2) Compatibility with telehealth (e.g., do not require laboratory-based equipment or highly trained clinical examiners).
(3) Adaptability for caregiver-facilitated administration (e.g., do not require specific training or multiple examiners).
(4) Suitability for children with severe developmental, motor, and speech/language delays.

We are in the process of developing additional tasks and adaptations for new samples, including older participants, children with specific genetic syndromes, and children with distinct medical needs (e.g., visual impairments). To facilitate scalability and data sharing, all tasks are archived and updated as relevant using our Open Science Foundation (OSF) web site.^1^

### PANDABox Development and Beta Testing

PANDABox was developed through an iterative process of consultation with field and local experts and simulated remote assessments with children with and without disabilities. The first four beta assessments were conducted live (in the laboratory setting with examiner present) with children with Down syndrome (14–27 months) to refine initial task selection. Next, the battery was piloted using simulated remote assessment in local children without Down syndrome (*n* = 20; 4–20 months). During this phase, participants completed the PANDABox battery in a separate room from the examiner. The examiner then returned to debrief the participant and conduct supplemental validation testing in-person. The finalized battery was then piloted in three children with Down syndrome (8–22 months) using simulated remote assessment to ensure tasks and adaptations were appropriate for children with disabilities. Over the course of these assessments, we adjusted iteratively the battery to optimize the clarity of instructions, data quality, and accessibility across ages. The battery was then “locked” for further major modifications for the purpose of the present study.

### Participants

Participants were 16 infants with Down syndrome ages 5–19 months (*M* = 11.9, *SD* = 3.9) and their caregivers, all of whom were biological mothers (28–43 years; *M* = 34.5, *SD* = 4.8). Families were recruited nationally via Facebook support groups. Inclusion criteria required that participants provide a documented medical diagnosis of Down syndrome, live in the continental United States, live in a home where the primary language was English, and have access to high-speed internet. As depicted in Table 1, the sample was limited in diversity and was primarily white, with most caregivers reporting income over $75,000 and completing higher education. Families lived an average of 676 miles from the host laboratory. Children’s adaptive behavior on the VABS-3 Parent Interview (Sparrow et al., 2016) was generally in the moderately low range, with Adaptive Behavior Composite scores ranging from 68 to 100 (*M* = 82.0, *SD* = 8.4).

**TABLE 1:** Demographic data.

Demographic feature	*N* (%)
**Race**	
White	13 (82%)
Asian	1 (6%)
Not reported	2 (13%)
Hispanic ethnicity	4 (25%)
Not reported	2 (13%)
Female sex	6 (38%)
**Household income**	
0–$15,000	1 (6%)
15,001–$35,000	0 (0%)
35,001–$75,000	3 (19%)
75,001–$150,000	3 (19%)
Over $150,000	4 (25%)
Not reported	5 (31%)
**Maternal education**	
Less than high school	0 (0%)
High school	0 (0%)
Some college	2 (13%)
2 years degree	1 (6%)
4 years degree	5 (31%)
Professional degree	5 (31%)
Doctorate	1 (6%)
Not reported	2 (13%)

### Materials

#### PANDABox

The PANDABox kit included a (1) Microsoft Surface Go Pentium Gold 4415Y 1.6 GHz 8 GB 128 GB, (2) two Actiwave Cardio monitors (CamNtech Inc., Boerne, TX, United States), (3) Logitech C525 HD web cam, (4) two LENA vocal recorders (Xu et al., 2008) and (5) module task materials.^2^ Materials for each kit cost $4,915. Caregivers also used their mobile or landline phone to stay connected with the examiner throughout the assessment, enabling the examiner to help them setup the computer and troubleshoot any connectivity issues.

#### Computer Preparation

The Surface Go computer was selected due to its low cost, high power for quality teleconferencing, and simple interface. The computer was installed with the following software: TeamViewer (TeamViewer GmbH, Göppingen, Germany), Microsoft Office, and Actiwave 2.0.8 heart rate monitor programming software (CamNtech Inc., Boerne, TX, United States). To maximize ease of use, computers were prepared in advance by removing all icons from the desktop except Google Chrome, Actiwave, TeamViewer, caregiver prompt PowerPoints, and consent forms. We also disabled automatic updates and time-zone synchronization to ensure software compatibility and facilitate subsequent integration of data streams.

#### Teleconferencing Software

After piloting several programs, we selected TeamViewer, a secure teleconferencing and remote connection software that uses 256-bit encryption and two-factor authentication. TeamViewer supports HIPAA-grade security and permits both live video chat *and* remote connection. Remote connection permits the examiner to control the computer from another site, enabling the caregiver to instead focus on implementing activities and managing their child’s behavior. For example, the examiner was able to control all basic functions of the computer (e.g., change volume, start and stop recordings), display stimuli, and troubleshoot challenges. TeamViewer also includes a user-friendly interface and requires minimum setup by families prior to each call.

#### Module Task Materials

Materials necessary for each specific module are summarized on our OSF site. In brief, each PANDABox included several sets of toys arranged in brightly colored and clearly labeled mesh bags to facilitate quick and easy identification by caregivers during the assessment. Toys were selected to be compact, durable, lightweight, and easy to clean.

#### Host Laboratory

The host laboratory (location of researcher teleconferencing) was equipped with a desktop computer (Dell OptiPlex 5050) equipped with TeamViewer and a webcam, and a landline phone. The laboratory was in a quiet, secure location.

### Measures

We administered a series of tasks designed to assess a wide array of behaviors relevant to early clinical risks. Tasks are detailed on our OSF site. A subset of tasks were adapted from the Lab-TAB (Goldsmith and Rothbart, 1996). In brief, tasks included (1) passive viewing of a children’s video to solicit sustained attention (“Attention: Video”; Tonnsen et al., 2018), (2) independent play with a variety of age-appropriate toys designed to capture developmental hierarchy of play skills (“Developmental Play”; adapted from McDuffie et al., 2015), (3) a series of caregiver-facilitated discrete presses designed to solicit autism-related symptoms (“Discrete ASD Presses”; described below), (4) an experimental press for frustration in which the caregiver prevents access to an engaging toy (“Negative Affect”; Lab-TAB), (5) an experimental press for positive engagement in which the caregiver presents the child with bubbles (“Positive Affect”; Lab-TAB), (6) independent play with a single toy to solicit sustained attention (“Attention: Toy”; Lab-TAB), (7) a 10-min unstructured parent–child interaction (“Parent–Child Interaction”; adapted from Freeman and Kasari, 2013) and (8) caregiver reading the child a provided book (“Parent–Child Story Time”; added after beta testing phase as additional measure of caregiver-child engagement that would be minimally impacted by severe child motor delays).

Additional manuscripts are underway to report the specific output of these tasks. Here, we focus on three primary indicators of whether administering these tasks via telehealth is feasible: (1) logistics and cost, (2) caregiver implementation fidelity and self-reported assessment experience and (3) child engagement and data quality.

#### Logistics and Cost

We examined rate of rescheduled and canceled appointments and estimated potential cost-savings of telehealth versus traditional administration of PANDABox. This analysis assumes recruitment of a national cohort of infants. Costs are estimated based on *n* = 6 (monthly rate of data collection for present study), *n* = 12, and *n* = 24 samples per month. Number of kits needed were estimated based on an 11-day shipping and repackaging window (i.e., downloading data, cleaning toys, repackaging kit) for telehealth-administration and a 4-day setup/repackaging window for traditional administration. Calculations include administration costs, participant costs (remuneration and lost wages), and travel.

We calculated cost for traditional assessments using an average trip duration of 2.5 days and 1.5 nights to account for variability in city of origin. We estimated airfare using 2019 average flight prices to our local airport ($362 × 2 passengers; United States Department of Transportation, 2019). To estimate lost caregiver wages for one caregiver due to travel assessments, we used the most recent (May 2018) national average annual wage of $51,960 (United States Department of Labor, 2018) adjusted to daily rate and multiplied by 1.6 to account for employer loss due to employee absenteeism (Nicholson et al., 2006). Calculations for traditional assessments do not include additional staffing necessary to plan and book travel or additional clinic space and project coordination staff needed for in-person assessments.

#### Caregiver Implementation Fidelity and Uptake

We measured caregiver responses to PANDABox using both self-report measures (pre- and post-assessment surveys) and objective coding (implementation fidelity). Self-report measures were added part-way through the study and were administered to 11 of 16 families.

##### Pre-assessment questionnaire

Caregivers completed a pre-assessment questionnaire so that the examiner could determine task adaptations and troubleshoot potential assessment barriers in advance (Table 2). The survey asked caregivers to rate their comfort with different types of technology and to predict how their child might respond to the telehealth-based assessment.

**TABLE 2:** Pre- and post-assessment questionnaires.

**Pre-assessment survey questions (*n* = 9)**				
How comfortable are you with…	Poor	Fair	Good	Excellent
Skype, Facetime, or other “videochat” technology	0	3	1	5
Tablet, iPad, or other touchscreen devices	0	1	4	4
Video camera setup and use	0	2	4	3
Learning new softwares or technology devices	0	1	5	3
Think of how your child behaves in his or her typical day-to-day routine in your home with you and or another caregiver. How similar or different do you think their behavior would look in the contexts below?	Very different	Somewhat different	Somewhat similar	Very similar
At the research lab with a research assistant	2	3	3	1
In your home with a research assistant	0	1	5	3
**Post-assessment survey questions (*n* = 10)**				
How satisfied were you with…	Poor	Fair	Good	Excellent
The voice quality of the assessment materials	0	0	0	10
The visual quality of the assessment materials	0	0	0	10
The ease of technology use	0	0	0	10
The ease of assessment materials	0	0	0	10
The length of time	0	1	1	8
Your personal comfort with the assessment	0	0	0	10
Your overall experience with the assessment	0	0	0	10
The clarity of instructions/directions	0	0	0	10
The amount of support from our team	0	0	1	9
The ability of our team to answer your questions	0	0	0	10
The sensitivity and friendliness of our team	0	0	0	10
The privacy of the assessment	0	0	0	10
The appropriateness of the assessment for your child’s abilities	0	1	2	7
The assessment’s ability to capture your child’s strengths	0	1	4	5
The assessment’s ability to capture your weaknesses	0	0	3	7
The assessment’s ability to capture your child’s typical behavior	0	3	2	5

##### Post-assessment survey

An online anonymous post-assessment survey was completed by caregivers directly after completion of the telehealth session (Table 2). The survey consisted of (1) 16 items assessing caregivers’ satisfaction with the telehealth session and (2) five open-ended questions about the positive and negative parts of the assessment, concerns with privacy, and any additional recommendations for future development.

##### Implementation fidelity

Implementation fidelity was coded offline by trained research assistants. We operationalized implementation fidelity as the degree to which, with standardized support, the caregiver implemented tasks to laboratory-based research standards. Coders rated caregivers’ implementation of 13 prompts as “ideal,” “sufficient,” or “poor.” Ideal and sufficient fidelity required the caregiver to follow the standardized directions with very few (ideal) to several (sufficient) deviations. Fidelity was considered poor when deviations were severe enough to result in data that would not be suitable for analysis. Fidelity was rated as “other” when tasks were not administered or obscured from the camera view. Each video was coded by two independent raters, with all disagreements resolved by an additional consensus coder. To train, coders (1) completed a brief training on study procedures and technology, which included reading administration materials and a coding manual, (2) coded two beta participants to reach over 80% agreement across tasks with a predetermined expert rater, and (3) independently coded files, with each file coded by two coders. Percent agreement across paired raters and tasks was 93%, with Gwet’s AC1 (GAC), an alternate Kappa calculation appropriate for skewed data (Gwet, 2008) of 0.93, in the “almost perfect” agreement range (McHugh, 2012).

#### Data Quality and Validity

We describe data quality and descriptive patterns across three levels: clinical, behavioral, and spectral. Behavioral and spectral measures were selected to span the full assessment battery, enabling preliminary validation of whether tasks solicited expected changes in the child’s response. For example, we expected less engagement and faster heart rate during a task designed to solicit frustration, and greatest child vocalization during parent–child interaction.

#### Clinical Data: Autism Specific Presses

Our initial protocol measured clinical features of emergent ASD using a series of presses based on the literature. We are in the process of piloting a number of specific presses but focus here on four presses coded to date. These presses examine the child’s response to (1) hearing their name (“Name Call”), (2) an engaging social game initiated by the caregiver (“Peekaboo”), (3) abrupt changes in caregiver affect (“Still Face”), and (4) being directed to follow a caregiver’s point (“Joint Attention”). These tasks were inspired by a number of early ASD screening tools (Stone et al., 2000; Robins et al., 2001; Bryson et al., 2008; Lord et al., 2012) but were adjusted for caregiver administration. The examiner coached the caregiver to administer each press by providing verbal instructions and also displaying prompts on the caregiver’s computer. The examiner provided corrective coaching and feedback using standardized instructions.

Data were coded offline by two independent coders (1 primary coder, 1 reliability coder) using criteria developed specifically for caregiver-facilitated administration available on OSF. In brief, each press was assigned a code from 0 to 2, with 0 indicating an expected response relative to typical development, 1 indicating a questionable or subtle response, and 2 indicating an atypical response. If a press was administered but did not produce sufficient behavior to assign a code, it was coded 8. If a press was not administered or if it was administered, but technical issues (e.g., camera angles) obstructed the coder’s view of the child’s or caregiver’s behavior, the response was coded 9. Raters reached 75% agreement and a GAC Kappa value of 0.71 across the four presses, with lowest agreement on Still Face (Name Call: 1.00; Peekaboo: 0.64; Still Face: 0.46; and Joint Attention: 0.71). Due to the low interrater reliability of Still Face task, which likely reflected challenges coding this task from video, we are currently optimizing this portion of the protocol and do not describe results further. Percent agreement and GAC Kappa excluding Still Face task reached 81% and 0.81, respectively, across the remaining three presses.

##### Behavioral data: child engagement

We operationalized child engagement as the proportion of video frames in which the infant appeared to be oriented to the assessment task. Raters assigned one of three codes at 5s intervals across the full duration of the assessment: “engaged,” “disengaged,” or “obscured.” Ratings were assigned based on still frame images. “Engaged” was operationalized as touching or looking at any telehealth materials or the infant’s caregiver, and “disengaged” was operationalized as touching or looking at other things in the infants’ environment or, for children with advanced motor skills, actively attempting to escape the tasks. Coders (1) completed a brief training on study procedures and technology, (2) coded two beta participants to reach over 0.80 GAC Kappa with a predetermined expert rater (observed GAC = 0.84), (3) independently coded the remaining 16 files, with the first three files independently coded by both coders to ensure reliability. Raters demonstrated 83% agreement and a GAC Kappa value of 0.78 across “engaged,” “disengaged,” and “obscured” codes. They also demonstrated high agreement (89%) and GAC Kappa (0.85) in identifying which portions of files were not able to be coded (e.g., engaged and disengaged versus obscured).

##### Behavioral data: vocalizations

Child vocalizations and adult words were measured using the LENA system (Xu et al., 2008) a small recorder worn by the child to capture the child’s vocalizations and other sounds or language occurring within the child’s immediate environment. The LENA software uses algorithms to classify recorded sounds as target child speech, other child speech, adult speech, electronic noise, or silence. Additional algorithms generate counts of target child vocalizations, adult words, and conversational turns between the target child and an adult in their immediate environment. These counts are then used to calculate norm-referenced metrics indicating how the child’s language environment compares to those of similarly aged peers. The LENA system also produces a file that includes timestamps for each sound event during the recording that can be used to summarize the sounds occurring during specific segments of the recording which enabled us to synchronize and integrate multiple data streams during secondary data processing.

Participants were asked to complete two LENA recordings: one full-day recording, and one brief recording *during* their PANDABox session. For the full-day recording, caregivers were instructed to place the recorder on their child when the child first woke up in the morning, and to leave the recorder on for at least 12 h. For the assessment-day recording, the examiner instructed the child’s caregiver to place the recorder on the child before PANDAbox tasks began, and the child’s caregiver removed and turned off the recorder at the end of the session.

##### Spectral data: heart rate

We recorded both caregiver’s and child’s heart activity throughout the remote assessment; for this paper, we focused on the child’s heart activity. Heart activity was measured using the Actiwave Cardio monitor, which records a single channel of ECG waveforms using two standard ECG electrodes that are attached to the chest with standard ECG pads. Prior to shipping the PANDABox to the family, the Surface Go computer was used to program the Actiwave Cardio monitors to begin data acquisition 30 min before the scheduled remote assessment at a sampling frequency of 1,024 Hz,^3^ permitting approximately 5.5 h of continuous recording.

Once the PANDABox was returned, research staff downloaded ECG data and obtained IBI data by: (1) visually inspecting raw ECG data in EDFbrowser (Version 1.67; Van Beelen, 2019) to determine the presence and quality of ECG waveforms; (2) segmenting raw ECG data into 10-min segments using in-house scripts to facilitate processing; (3) manually coding and marking ECG fiducial points (i.e., R- or S-waves) in QRSTool (Version 1.2.2; Allen et al., 2007); (4) visually verifying marked ECG fiducial points in QRSTool; (5) merging marked ECG data using in-house scripts to obtain IBI data; and (6) editing IBI data in CardioEdit (2007). Version 1.5; Brain Body Center for Psychopsysiology and Bioengineering to correct for remaining artifacts.

#### Secondary Data Processing

To segment behavioral (child engagement and vocalization) and spectral (heart rate) data into tasks, coders used ELAN (Version 5.7; Max Planck Institute for Psycholinguistics, 2019) to mark (1) the start and stop times of each task and (2) the Surface Go computer time pictured in the behavioral video recording. We then used in-house data processing scripts in SAS (Version 9.4; Sas Institute Inc., 2019) and R (Version 3.5.3; R C Team, 2019) to align behavioral and spectral data streams and segment the integrated data by task.

### Procedures

Once participants completed screening and informed consent, caregivers were asked to complete the telehealth assessment, VABS-3, and LENA full-day recording within a 1-week timeframe. Caregivers completed the pre-assessment questionnaire and a series of developmental rating scales approximately 1–2 weeks before the assessment. They were then mailed the PANDABox. Caregivers completed the LENA full-day recording either before or after the telehealth assessment, depending on their availability.

On the day of the assessment, the examiner called the caregiver and stayed on the phone throughout the assessment to provide continuity in the case of any technological problems. The examiner began by instructing the caregiver to log onto the tablet, connect to Wi-Fi, and run TeamViewer. After obtaining consent to continue with the telehealth session, the examiner instructed the caregiver on setting up the remaining technology for the assessment, including applying heart rate monitors, starting the LENA recording, and plugging in the webcam. The examiner then started the video recording and supported the caregiver and participant to complete the core tasks. Across the assessment, the examiner either narrated or asked the caregiver to use the web cam to capture the Surface Go computer time in the behavioral video recording, which enabled synchronization and integration of multiple data streams during secondary data processing. Following the final task, the examiner instructed the caregiver to remove the heart rate monitors and stop the LENA recording. Before exiting TeamViewer, the examiner stopped the video recording, discussed PANDAbox pickup, and opened the post-assessment survey on the participant’s tablet. The participant was asked to complete the survey when the examiner logged off of TeamViewer to preserve their confidentiality. To return the kit, the caregiver placed a return shipping label, provided by study staff, on the kit and left the kit outside of their front door to be picked up by the United Parcel Service.

## Results

**FIGURE 1:**
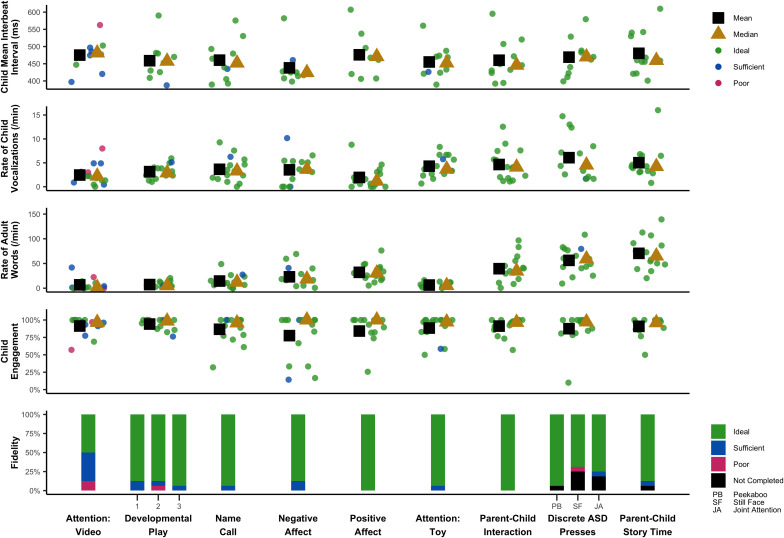
Sample clinical, behavioral, and spectral outcome variables across PANDABox tasks.

This study was primarily descriptive in nature, with additional projects underway to characterize task performance in larger samples. Here, we descriptively summarize key trends in the data. These data are summarized in a single time-series in Figure 1, which displays key fidelity, engagement, behavioral, and spectral data across the session.

### Logistics and Cost

#### Scheduling

All assessments were successfully completed. Consistent with the flexibility afforded by telehealth, participants could reschedule sessions if needed. Of 16 reported assessments, two were rescheduled for changes in the caregiver’s work schedule (*n* = 1) or changes in the infant’s sleep schedule (*n* = 1). Four additional assessments were paused or moved slightly to accommodate changes to the infants’ schedules (e.g., unexpected changes in naps and feedings). No kits or equipment were lost or damaged in transit. Two families not included in this manuscript scheduled an assessment but were unable to participate due to unexpected medical problems (*n* = 1) or lack of time due to other family commitments (*n* = 1).

#### Cost-Benefit Analysis

Estimated cost-savings for PANDABox versus traditional administration is detailed in Table 3. Relative costs did not change at a linear rate, as we rounded kit numbers to the nearest whole number but decreased with volume. Relative to traditional assessments, telehealth administration was estimated to be more expensive for the smallest sample (*n* = 6; $763 monthly difference; 5%) but less expensive for *n* = 12 ($9,939; 30%) and *n* = 24 ($12,551; 20%). Telehealth-based data collection was cost-saving for rates of seven participants/month ($1,020; 6%) or greater. These estimates do not include facilities and administrative cost-savings, as described previously, thus likely underestimate true savings.

**TABLE 3:** Cost-benefit analysis.

	Current study	Projected sample sizes	
Monthly sample volume	6	12	24
**Telehealth administration**			
*Administration costs*			
Researcher computer	$686	$686	$686
Team viewer	$950	$950	$950
PANDABox kits needed	3	4	9
Kit costs	$14,745	$19,660	$43,368
Shipping (2-way test kit; $84)	$504	$1,008	$2,016
*Participant costs*			
Remuneration ($55)	$330	$660	$1,320
**Total cost of telehealth**	$17,215	$22,964	$48,340
**Traditional administration**			
*Administration costs*			
PANDABox kits needed	1	2	3
Kit costs	$4,915	$9,830	$14,745
Shipping (1-way LENA; $15)	$90	$180	$360
*Travel costs*			
Two flights ($361 each)	$4,332	$8,664	$17,328
Baggage ($60)	$360	$720	$1,440
Airport parking ($15/day)	$225	$450	$900
Lodging ($95.09/night)	$856	$1,712	$3,423
Meals ($55.24/day each)	$1,657	$3,314	$6,629
*Participant costs*			
Caregiver lost wages ($142.36/day)	$3,417	$6,833	$13,666
Remuneration ($100)	$600	$1,200	$2,400
**Total cost of traditional**	**$16,452**	**$32,903**	**$60,891**
**^a^Telehealth cost savings**	($763)	$9,939	$12,551
**Per participant**	($127.24)	$828.26	$522.97

*^a^For traditional administration, we estimated costs based on a trip duration of 2.5 days and 1.5 nights to account for 1–2 nights of lodging and 2–3 days of per diem expenses, depending on city of origin. Travel and caregiver wage costs were estimated using government-reported rates for our local region.*

### Caregiver Implementation Fidelity and Uptake

#### Pre-assessment Questionnaire

Of the 11 caregivers who were sent questionnaires, nine were completed. The majority of caregivers described “good” or “excellent” comfort with video chat technology, touchscreen devices, video camera setup, and learning new technologies (Table 2). No caregivers indicated “poor” comfort levels with technology. When asked to predict how their child’s typical day-to-day behavior would be impacted by participating in research in laboratory versus home settings, five indicated their child would act “somewhat” or “very” differently at a research lab, whereas only one anticipated different behavior in a home-based research setting. In other words, most families anticipated their child would behave typically in home-based, but not clinic-based, research protocols.

#### Post-assessment Survey

Of the 14 caregivers who were sent anonymous post-assessment surveys, 10 were completed. Quantitative survey data (Table 2) suggested positive experiences, with 97% of responses rated as “good” or “excellent” and 0% of responses as “poor” across items related to satisfaction with assessment materials, ease of technology, duration, examiner support, and privacy. Slightly lower satisfaction was reported for the assessment’s ability to capture the child’s typical behavior, with 7 of 11 “good” or “excellent” responses. Qualitative data paralleled these findings and are available on OSF.

#### Implementation Fidelity

As detailed in Table 4, implementation fidelity was acceptable (“Ideal” or “Sufficient”) for 94% of coded tasks (Ideal: 86%, Sufficient: 8%, Poor: 2%, Other: 4%). “Poor” fidelity was most common for the Attention: Video task (12.5%) and was infrequent (<5%) across all other tasks. Implementation fidelity was similar for caregivers who self-reported “fair” comfort with technology on any of the prescreen items (*n* = 3; Ideal/Sufficient: 97%, Poor: 3%) versus those who indicated consistently “good” or “excellent” comfort with technology (*n* = 6; Ideal/Sufficient: 99%; Poor: 1%).

**TABLE 4:** Caregiver implementation fidelity and child engagement.

Task	Caregiver implementation fidelity (%)				Child engagement (%)		
	Ideal	Sufficient	Poor	Other	*M* (*SD*)	Minimum	Maximum
Attention: video	50	37.5	12.5	0	91 (13)	57	100
Developmental play	89.6	8.3	2.1	0	94 (8)	76	100
*Name call	93.8	6.2	0	0	87 (19)	32	100
Negative affect	87.5	12.5	0	0	78 (33)	14	100
Positive affect	100	0	0	0	84 (29)	0	100
Attention: toy	93.8	6.2	0	0	88 (17)	50	100
Parent–child interaction	100	0	0	0	91 (12)	57	100
*Discrete ASD presses	79.2	2.1	2.1	1.6	88 (23)	10	100
Parent–child story time	87.5	6.2	0	6.2	91 (14)	50	100
Overall	86.1	7.7	1.9	4.3	91 (9)	72	99

*Additional play-based presses were piloted in a portion of participants, focused on constructs such as stereotyped behaviors, response to changes in facial affect, and social smiles. These presses are included in engagement data but excluded in fidelity data.

### Data Quality and Validity

#### Clinical Data: Autism Specific Presses

Across the three sample tasks, 73% of tasks were completed and coded. Reasons for missing task data included insufficient amount of behavior to code the press (code = 8: 19%) and obstructed camera view or missing administrations (code = 9: 8%). Seven participants missed 1 task, three participants missed 2 tasks, and no participants missed all tasks. For Name Call (*n* = 12), 25% of children responded to their name on any of the three calls (code = 0), 25% demonstrated a subtle response, such as pausing their play or making a vocal response (code = 1), and 50% did not respond to their name by making eye contact with their caregiver on any call (code = 2). For Peekaboo (*n* = 13), 77% of children showed enjoyment and some indication of wanting the game to continue (code = 0), 8% showed enjoyment with no indication of wanting the game to continue (code = 1), and 15% did not demonstrate engagement or enjoyment while their caregiver played peekaboo with them (code = 2). For the Joint Attention task (*n* = 10), 80% responded by turning their head toward where their caregiver was pointing (code = 0), and 20% either looked at their caregiver’s face or her finger as she pointed (code = 1). No children demonstrated a lack of response (code = 2). Thus, we were able to solicit and code variable behavior from caregiver-facilitated ASD presses.

#### Behavioral Data: Child Engagement

Child engagement was coded for 97% of the data; 3% could not be coded because the infant was out of view, the still frame was too blurry, or engagement could not be discerned for any other reason. Infants were rated as engaged for 91% of the coded data (Table 4). Most infants remained on task across the entire assessment, with only one infant falling below 75% engagement (Figure 1). The Negative Affect task demonstrated relatively lower engagement, which is expected given the task specifically solicits frustration. The Negative Affect, Positive Affect, and Discrete ASD presses appeared to demonstrate higher variability in child engagement compared to other tasks.

#### Behavioral Data: Vocalizations

Language ENvironment Analysis data were missing for 1 of 16 home recordings and 1 of 16 assessment recordings due to data transfer issues. Daylong recordings (*M* = 13.69 h; *SD* = 2.21) produced LENA standard scores of 83–130 for child word count (*M* = 101.50, *SD* = 14.01) and 81–150 for adult word count (*M* = 105.58, *SD* = 20.58). The average rate of vocalization was 99 vocalizations per hour for the child and 1,075 words per hour for adults in the child’s environment. LENA standard scores are not available for short-term recordings due to software restrictions, however, *post hoc* calculations using raw data indicated rates of 227 vocalizations per hour for the child and 1,668 words per hour for adults; thus, vocalization rates were higher during PANDABox than average rates across a typical day. Rate was generally consistent across tasks, with the exception of a generally lower rate during the Positive Affect task. Within each task, there was also a great amount of variability in child vocalization rate between children. Adult word rate varied across tasks, with rate being lowest during Attention: Video, Developmental Play, and Attention: Toy, and highest during Parent–Child Story Time, Parent–Child Interaction, and Discrete ASD Presses. These patterns of adult word rate are expected based on the nature of the task and the caregiver’s instructed involvement.

#### Spectral Data

Electrocardiograph data were available for 12 of the 16 participants with a mean duration of 66.63 min (*SD* = 31.60). ECG data was unavailable for one participant due to a rescheduled assessment that did not align with the original programming of the Actiwave Cardio monitor. Three participants had unusable data that consisted of high frequency noise. Two of the remaining 12 participants had limited ECG data due to an unintended but preventable programming issue for the Actiwave Cardio monitors that did not account for daylight saving time change. Less than 0.29% (*M* = 0.07%, *SD* = 0.09%) of each participant’s IBI data had to be edited, indicating the feasibility of collecting high-quality ECG data in remote assessments and the effectiveness of our data processing pipeline.

Overall, the IBI data revealed substantial between- and within-participant variation across tasks and appeared to reflect cardiac fluctuations consistent with general principles of physiological functioning. For example, for five (50%) participants with IBI data across all tasks, the smallest mean IBI (i.e., fastest heart rate) occurred during Negative Affect, which was a task where bodily struggles and distress vocalizations were typical responses to prohibited access to an attractive toy; in contrast, for five (50%) participants with IBI data across all tasks, the largest mean IBI (i.e., slowest heart rate) occurred during Attention: Video or Parent–Child Story Time, which were passive tasks that involved visual and auditory attention. Together, these trends suggest our protocol is soliciting and measuring expected changes in heart activity across experimental presses.

## Discussion

The present study provides initial evidence that home-based, remotely administered laboratory sessions are possible for researchers studying infants at risk for ASD, including those with NGS. The PANDABox protocol was (1) well received by both caregivers and infants, (2) required low resources to administer, and (3) generated high quality, integrated clinical, behavioral, and spectral data. These data suggest that with further development, low-cost laboratory-grade research protocols may be remotely administered by caregivers in the family home, opening a new frontier for cost-efficient, scalable assessment studies for children at risk for ASD and other neurodevelopmental disorders.

### PANDABox Was Well-Received by Families

The PANDABox protocol exhibited a number of strengths, including favorable caregiver uptake and satisfaction and strong implementation fidelity (94% acceptable data). These data suggest that– consistent with the substantial literature base on parent-facilitated telehealth interventions (Vismara et al., 2013; Nelson et al., 2018)–caregivers are capable of administering simple, examiner-guided assessment tasks with high fidelity. Caregivers responded positively to the telehealth assessment experience, with 97% of anonymous post-assessment survey data indicating “good” or “excellent” satisfaction across various components of the assessment, including technical support, examiner behavior, ease of use, and privacy, again paralleling recent work (Talbott et al., 2019). Although much of the literature on caregiver experiences in assessments have focused on the initial diagnostic appointment, more recent work in NGS suggests that many caregivers would prefer to be more directly involved in their child’s repeated clinical and research assessments that follow the diagnostic appointment, potentially reflecting that for rare NGS in particular, caregivers’ expertise about their child’s condition and individual needs may exceed that of the provider (Kelleher et al., 2020). Importantly, a number of recent studies have suggested that parents are capable of implementing assessment and treatment tasks with fidelity, with some studies even suggesting slight benefits of parent-facilitated treatment outcomes in home- versus clinic-based settings (Lindgren et al., 2016). As such, enabling families to actively engage in the assessment process may alleviate some of the negative experiences commonly reported by families of children with neurodevelopmental disorders (Casey et al., 2012; Kelleher et al., 2020) support rigor and reproducibility, and increase the engagement of caregivers and children in research studies and trials.

Caregiver fidelity data also provides us with preliminary guidelines for further optimizing PANDABox to maximize participant comfort and data quality. In our small sample, fidelity did not appear to be impacted by caregivers’ self-reported comfort with technology, although our sample was also predominantly highly educated and therefore may still have greater technical skills than the general population, necessitating additional research in this area. Fidelity data did, however, vary slightly across assessment tasks, with relatively lower scores on the Attention: Video task. This task occurred first, suggesting that a “warm-up” period may be needed to help caregivers feel comfortable with the assessment process. It is also possible that the current instructions need to be optimized. Regardless, this variability highlights that as PANDABox expands to new tasks and participant samples, continuously monitoring and optimizing based on fidelity is critical to ensuring effective and user-friendly implementation. Notably, during the Attention: Video task, one infant of a caregiver with “poor” fidelity exhibited the most atypical heart activity, engagement, and vocalization rate (Figure 1), suggesting fidelity data may provide useful context for conceptualizing variability across individual participants.

In addition to favorable uptake by caregivers, infants also generally cooperated throughout the assessment process, remaining on task for over 90% of the assessment. Data loss due to infant fussiness or fatigue is a common challenge for researchers and has been described as a challenge facing reproducible developmental science (Frank et al., 2017). Although we did not compare engagement in PANDABox and laboratory-based protocols directly, the majority of participants (7 of 10) reported that PANDABox’s ability to capture their infants’ typical behavior was “good” or “excellent” (“fair”: *n* = 3, “poor”: *n* = 0). Of course, research protocols are unlikely to truly capture naturalistic infant behavior, often by design –the use of equipment, experimental manipulations, unfamiliar staff members, and new activities may all impact how a child responds in-session. However, for research to be generalizable, it is important to be able to accurately estimate this margin of difference and how it might vary across participants. For example, our preliminary data suggest that average rate of child vocalization is over twice as high during PANDABox compared to full-day recordings. A number of factors may produce this increase, including the interactive, play-based nature of PANDABox tasks and the likelihood that full-day recordings include periods of inactivity and naps. However, to approximate the margin of difference between behaviors during the assessment battery and everyday activities, we are currently conducting follow-up studies to examine the “active ingredients” that solicit different infant responses across a variety of domains. For example, we will attempt to quantify the impact of telehealth (PANDABox versus live), location (laboratory and home) and examiner (caregiver and researcher), paralleling similar efforts in the developmental field (Frank et al., 2017). This work will inform the interpretation of telehealth-based data by quantifying the potential impact of uncontrolled tertiary variables on infant performance.

### PANDABox Required Few Resources to Administer

The PANDABox assessment was also inexpensive to administer relative to the potential costs associated with clinic-based assessments of rare patient populations. Indeed, telehealth-based research has been recognized for potential cost-saving benefits, although few studies have specifically quantified the relative financial benefits of this approach. For the present study, PANDABox materials relied primarily on commercially available software and equipment and easily sourced play materials. We are currently exploring a number of lower-cost adaptations. However, using the current kit costs, we estimated that telehealth-based PANDABox administration to a national cohort would be more cost-effective for data collection rates of seven participants/month or greater. Notably, these cost-saving benefits do not account for the increased administrative and facilities burden associated with in-person assessments, potentially underestimating the true savings potential of telehealth-based administration. Given our sample was generally quite affluent, we expect additional costs will emerge when extending PANDABox to lower resource communities, such as purchasing WiFi “hotspots” for families with no internet access or replacing kits that are lost or stolen in transit. As we extend PANDABox to new communities, additional research is needed to find the optimal balance between equipment cost and data quality.

### PANDABox Generated High Quality, Integrated Data

One of the key take-home findings of this study is that clinical, behavioral, and spectral data can be collected remotely and integrated with commercially available technology. Across tasks, rates of data loss were generally low, with only 3% of video frames omitted from child engagement coding because the child was not in the frame or the image was blurry. Rates of data loss were similarly low for LENA recordings (6%) but were higher for ECG (25% fully missing, 13% partially missing) and discrete behavioral presses (27%), reflecting the increased technical demands for these activities. Some data loss was unanticipated but preventable (e.g., misprogramming heart monitors across daylight savings time), suggesting these rates will improve in the future. Other groups have reported similarly elevated rates of missing spectral data, with at least one major study in autism reporting unusable wearable device data in their initial protocol (Ness et al., 2019) suggesting an area of need in the field. Data loss for the discrete behavioral presses were more frequently due to having insufficient amount of behavior to code (19%) rather than technical issues (8%), which suggests that caregivers may benefit from additional supports such as concrete video examples of administration and opportunities to administer the presses during naturalistic rather than “on-demand” portions of the telehealth session. Efforts are underway to continuously adapt and optimize the caregiver and examiner instructions to maximize data completeness and integrity.

### Future Directions and Limitations

As PANDABox is scaled to new populations and research teams, we are developing methods to promote high quality, integrated data collection. Specifically, we have developed an OSF^4^ website to store up-to-date manuals, processing instructions, and disseminated data, which is accessible to approved researchers. Our intent is to create a community for which PANDABox can provide a platform for multiple research teams to collect compatible data while still retaining the freedom and flexibility to add and adapt tasks for novel use. To do so, we have created several guidelines for PANDABox collaboration, including expectations that all PANDABox users (1) implement the GUID system to ensure data can be collapsed across sites, (2) register use of PANDABox with our team, enabling us to track which projects are using which tasks, (3) register new tasks and task modifications with our team, enabling us to archive the evolution of the battery, and (4) link all disseminated findings to the primary PANDABox site on OSF. Although the nature of the PANDABox community and expectations for communal use will undoubtedly evolve with time, these initial steps will ensure the platform can facilitate high-powered, collaborative science across diverse neurodevelopmental research teams.

An important next step will be customizing PANDABox for families from diverse communities, including global populations and domestic families with lower literacy skills, financial resources, or English fluency. Indeed, a major limitation of our current study was lack of diversity, as our caregivers were mostly white, affluent, and highly educated. This bias may stem from our recruitment strategies, which predominantly relied on Facebook and registries that may favor families already well-integrated into virtual support networks and those who are have time and interest in participating in research. However, the presence of such pronounced bias in even our small pilot study reflects that telehealth alone is unlikely to substantially reduce health disparities in clinical trials. Indeed, a recent study of patient-reported barriers in diverse communities identified resource and time constraints as one of many factors limiting participation, with other barriers including general mistrust, lack of comfort with the research process, limited information about the study, and lack of awareness of how participation can benefit the patient and society (Clark et al., 2019). As such, addressing the severe health disparities in clinical trials – and the outcome measures used in such trials – will require a wholistic approach that goes beyond simply reducing geographic barriers alone.

A final next step will be to enhance training materials and procedures for PANDABox, facilitating scalability through parallel data collection procedures across sites. In the context of interventions, Glasgow et al. (2012) have proposed five key values of implementation science: (1) rigor and relevance, including attention toward external validity across diverse communities, (2) efficiency and speed, including leveraging shared databases to accelerate scholarship on variability of outcomes, (3) collaboration through team science, (4) improved capacity, including novel training solutions such as webinars, and (5) cumulative knowledge through documented progress in centralized, public forums. Many of these values are reflected in our current PANDABox methods, including the use of OSF and emphasis on a growing team science community. However, as we move forward with extending PANDABox into broader research spaces, we aim to strike an important balance between promoting rigor and reproducibility and maximizing access to PANDABox, particularly for low-resource laboratory teams and early stage investigators. Indeed, the commercialization of proprietary ASD assessment tools and training has been blamed, in part, for persistent global disparities in ASD services, as existing tools require high per-use fees, rigorous paid trainings, and adaptation fees (Durkin et al., 2015). Avoiding this propriety model will require creative solutions, such as use of electronic training or peer-to-peer consultation. Substantial research will be needed to ensure training protocols are accessible yet rigorous, enabling data to be collapsed across groups and meaningfully shared for common good.

## Conclusion

The present study provides an initial step toward telehealth-based laboratory experiences in the field of neurodevelopmental disorders. Our findings suggest that with support, caregivers are capable of collecting high-quality, research-grade data with minimal data loss. Relative to clinic-based administrations that require travel, telehealth-based PANDABox administration was projected to be more cost effective at a modest rate of assessment. Moreover, we were able to integrate clinical, behavioral, and spectral data offline, providing a nuanced framework for investigating multiple layers of responses – such as psychophysiological and high-density time-series data – that are more complex than typically assayed in telehealth-based research. Looking forward, our goal is to further enhance the applications and scalability of PANDABox, potentially providing a cost-effective, open science platform for the research community to conduct laboratory grade assessments remotely. With proper optimization, our hope is that PANDABox may be particularly beneficial in connecting researchers and patients from underserved communities, including in rural areas. Although substantial development and validation will be needed to achieve this goal, PANDABox may provide a scalable method for collecting higher-powered, lower-cost, and more representative neurodevelopmental data from low-incidence clinical populations.

## Data Availability Statement

The datasets generated for this study are available on request to the corresponding author.

## Ethics Statement

The studies involving human participants were reviewed and approved by Institutional Review Board – Purdue University. Written informed consent to participate in this study was provided by the participants’ legal guardian/next of kin.

## Author Contributions

BK conceptualized the project, oversaw data collection, and drafted the manuscript. TH, WN, and LH designed and executed coding and data processing pipelines for fidelity and engagement (TH), heart rate analyses and data synchronization (WN), and vocalization and discrete autism press data (LH). NW led data collection and designed PANDABox materials and administration procedures. LA mentored BK on project design and telehealth-based research. All authors read and approved the final manuscript.

## Conflict of Interest

The authors declare that the research was conducted in the absence of any commercial or financial relationships that could be construed as a potential conflict of interest.

## Footnotes

1 https://osf.io/gh962/

2 For families of older children, we recommend also including a wireless headset with Bluetooth compatibility to minimize child distractibility and examiner interference.

3 The sampling frequency could be reduced to 512 or 256 Hz, permitting approximately 11 or 22 h of continuous recording, respectively.

4 http://osf.io

## Abbreviations

ASD: autism spectrum disorders
ECG: electrocardiograph
GAC: Gwet’s AC1
GUID: NIH Global Unique Identifier
IBI: interbeat interval
IDD: intellectual and developmental disabilities
Lab-TAB: Laboratory Temperament Assessment Battery
LENA: Language ENvironment Analysis
NGS: neurogenetic syndromes
OSF: Open Science Framework
PANDABox: Parent-Administered Neurodevelopmental Assessment
VABS-3, Vineland Adaptive Behavior Scales: 3rd edition.
